# Cortical Morphogenesis during Embryonic Development Is Regulated by miR-34c and miR-204

**DOI:** 10.3389/fnmol.2017.00031

**Published:** 2017-02-09

**Authors:** Morten T. Venø, Susanne T. Venø, Kati Rehberg, Jessy V. van Asperen, Bettina H. Clausen, Ida E. Holm, R. Jeroen Pasterkamp, Bente Finsen, Jørgen Kjems

**Affiliations:** ^1^Department of Molecular Biology and Genetics, Interdisciplinary Nanoscience Center, Aarhus UniversityAarhus, Denmark; ^2^Department of Translational Neuroscience, Brain Center Rudolf Magnus, University Medical Center UtrechtUtrecht, Netherlands; ^3^Neurobiology Research, Institute of Molecular Medicine, University of Southern DenmarkOdense, Denmark; ^4^Laboratory for Experimental Neuropathology, Department of Pathology, Randers HospitalRanders, Denmark

**Keywords:** brain development, embryonic development, neuronal migration, microRNA, cortical morphogenesis, miR-204, miR-34c

## Abstract

The porcine brain closely resembles the human brain in aspects such as development and morphology. Temporal miRNA profiling in the developing embryonic porcine cortex revealed a distinct set of miRNAs, including miR-34c and miR-204, which exhibited a highly specific expression profile across the time of cortical folding. These miRNAs were found to target Doublecortin (DCX), known to be involved in neuron migration during cortical folding of gyrencephalic brains. *In vivo* modulation of miRNA expression in mouse embryos confirmed that miR-34c and miR-204 can control neuronal migration and cortical morphogenesis, presumably by posttranscriptional regulation of DCX.

## Introduction

Over the course of evolution, the mammalian brain has grown, both in volume and surface. In higher mammals, including humans, this enlargement within the confinement of the skull has been enabled by increasing the folding and convolution of the cerebral cortex (gyration) leading to increased surface of the brain. In the mammalian brain, the cerebral cortex is in charge of complex cognitive functions (Geschwind and Rakic, 2013). Normal cortical development is initiated from a functional subdivision of progenitors resulting in multiple layers (lamination), or zones, within the cortex. Neural progenitor proliferation occurs mainly in the ventricular zone of the developing cortex. Here, a high level of asymmetrical cell division of radial glial cells occurs, resulting in both new radial glial cells and more differentiated daughter cells, including neurons (Sun and Hevner, 2014). Following this step, proper lamination is primarily ensured by regulated migration of neurons and intermediate progenitors, toward the pial surface of the cortex, closest to the skull. The development ends in the cortical plate, consisting mainly of fully differentiated neurons (Geschwind and Rakic, 2013; Sun and Hevner, 2014). In gyrencephalic species, one model proposes that regional differences in proliferation and growth of intermediate progenitors cause the ridge (gyri) and groove (sulci) appearance of the folded neocortex. While the regulatory mechanisms are currently unclear, neuronal migration-dependent lamination seems to be a prerequisite for normal cortical folding. Consequently, aberrant neuronal migration is associated with cortical deformities, in the most severe form a completely smooth brain surface (lissencephaly) (Reiner, 1999). A smooth brain surface can be naturally observed in lissencephalic species such as small rodents (mice and rats) (Sun and Hevner, 2014). Mutations causing lissencephaly in gyrencephalic species are associated with brain malfunctions including mental retardation and epilepsy (des Portes et al., 1998; Shimojima et al., 2010).

The first mutation identified in humans associated with lissencephaly was in a heterotrimeric G protein, which accordingly was named lissencephaly-1 (LIS1, also known as PAFAH1B1; Reiner et al., 1993). LIS1 mutation, duplication, or knockdown has deleterious effects on gyration. Due to a role in dynein and actin polymerization, LIS1, serves an important role in brain development affecting both proliferation of neuronal precursors and impacts migration of newly formed neurons within the cortex. An alternative cause of lissencephaly, X linked-1 (LISX1), is formed by mutations in doublecortin (DCX) (des Portes et al., 1998). DCX is a microtubule-associated protein (MAP), which stabilizes microtubule protofilaments and regulates actin cytoskeleton dynamics (Tsukada et al., 2005; Slepak et al., 2012). Knockdown of DCX *in utero* inhibits migration of cortical neurons (Kriegstein and Noctor, 2004). Consequently, based on these lissencephaly disease models a picture emerges supporting cytoskeleton arrangement and neuronal migration as essential components of normal brain development in gyrencephalic species. Interestingly, more than 90% of children suffering from lissencephaly experience epileptic seizures (Dobyns, 2010; Herbst et al., 2016) and abnormal cortical lamination caused by perturbations in neuronal migration is the likely cause of this high seizure susceptibility (Stouffer et al., 2016). Proper lamination is a complex process highly dependent on regulation on many levels, including posttranscriptional regulation of gene expression by microRNAs (miRNAs; Sun and Hevner, 2014). The implication of miRNAs is based on the observations that (1) cortex-specific knockout of the miRNA-processing enzyme Dicer causes reduced cortex size in mice, (2) neural progenitor specific knockout of Dicer results in reduced neural progenitor pool and impaired neuronal differentiation (Saurat et al., 2013), and (3) Dicer deletion in postmitotic neurons is associated with increased neuronal cell density in the cortical plate (Davis et al., 2008). Previous studies have found that miR-22 and miR-124 affect cortical neuron migration by targeting components of the coREST/REST complex, indirectly regulating DCX expression (Volvert et al., 2014). Also miR-9 has been shown to affect migration by targeting Stathmin, a protein that increases microtubule instability (Delaloy et al., 2010) or the transcription factor, Foxp2, in conjunction with miR-132 (Clovis et al., 2012). Finally, miR-128 was shown to affect migration by regulating PHF6 (Franzoni et al., 2015). However, these miRNAs are not sharply regulated at the time of cortical folding, suggesting that, although functionally important, they are not key regulators of the process. Mir-34a, a close relative of miR-34c, was recently found to play an active role in neural cell differentiation and was shown to affect migration of neuroblasts. The authors found DCX as a target for miR-34a, which is a likely contributor to the observed role for miR-34a (Mollinari et al., 2015).

We recently profiled the expression level of miRNAs in the porcine cerebral cortex, at five different timepoints across embryonic development (manuscript in preparation). A specific subset of miRNAs exhibited a highly distinct upregulation between day 60 and 80 post gestation (time of gyration). Here we unveil a functional role for two of these miRNAs in neuronal migration, miR-34c and miR-204. Combined with our discovery that these two miRNAs show extreme expression differences across cortical folding in the pig, suggests that they are instrumental in establishing the correct cortical morphogenesis in gyrencephalic animals.

## Results

### Differential expression of miRNAs in embryonic porcine cortex

**Figure 1:**
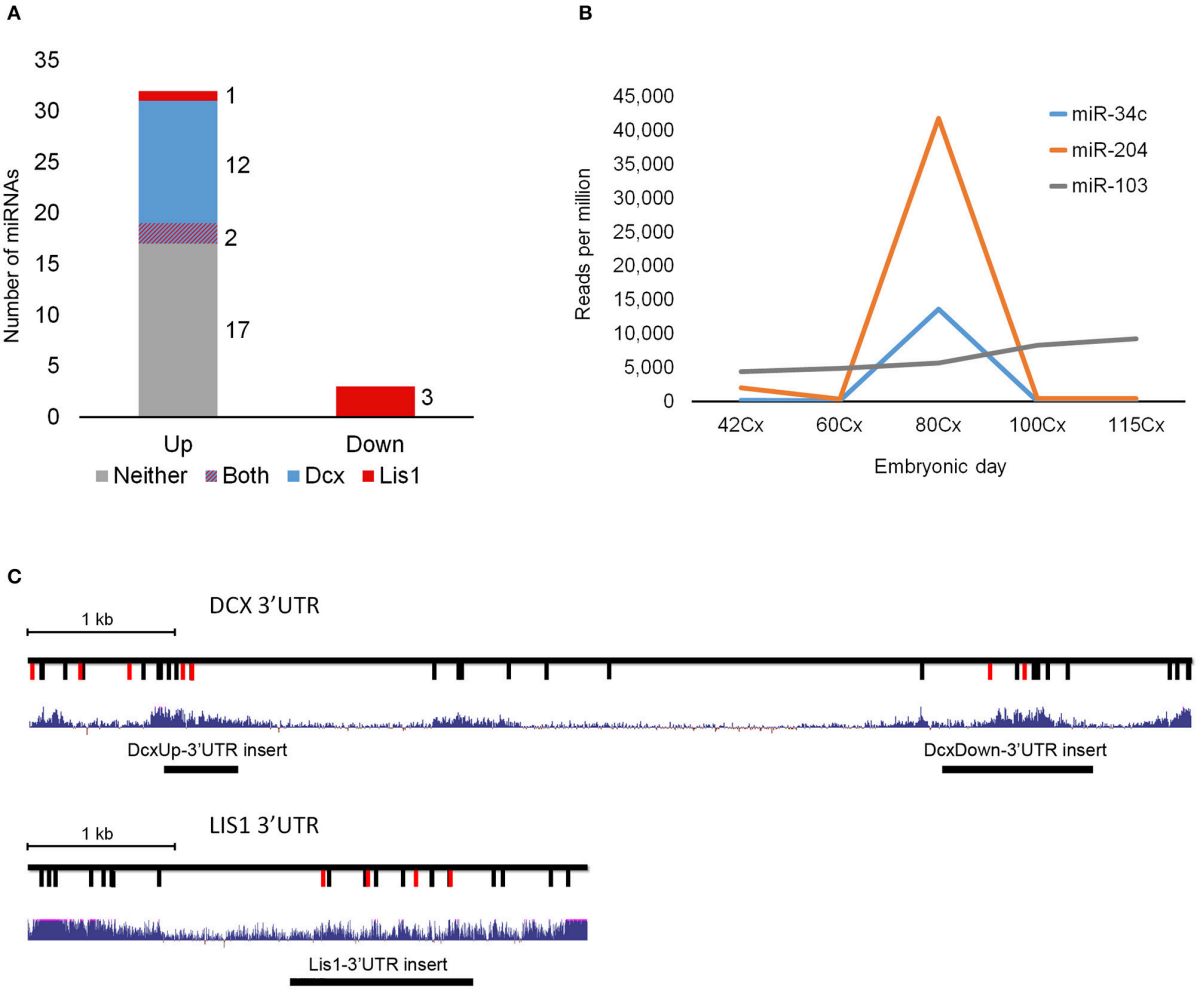
**DCX and LIS1 targeting miRNAs. (A)** The number of miRNAs up- or downregulated from E60 to E80 are shown. Color code indicates if differentially expressed miRNAs are predicted to target LIS1 (red), DCX (blue), both (red and blue), or neither (gray). **(B)** The RPM normalized expression of differentially regulated miR-34c and miR-204 as well as the stable miR-103. One replicate was used per timepoint. **(C)** Schematic illustrations of DCX and LIS1 3′UTRs with indication of conserved TargetScan7 predicted miRNA target sites. For DCX 3′UTR red vertical lines indicate target sites of upregulated miRNAs. For LIS1 3′UTR red vertical lines represent downregulated miRNA target sites. Black vertical lines indicate miRNA target sites for non-differentially expressed miRNA. Blue track shows UCSC Genome Browser's 100 vertebrates basewise conservation by PhyloP. Location of insert fragments used for luciferase reporter assay are indicated.

**Figure 2:**
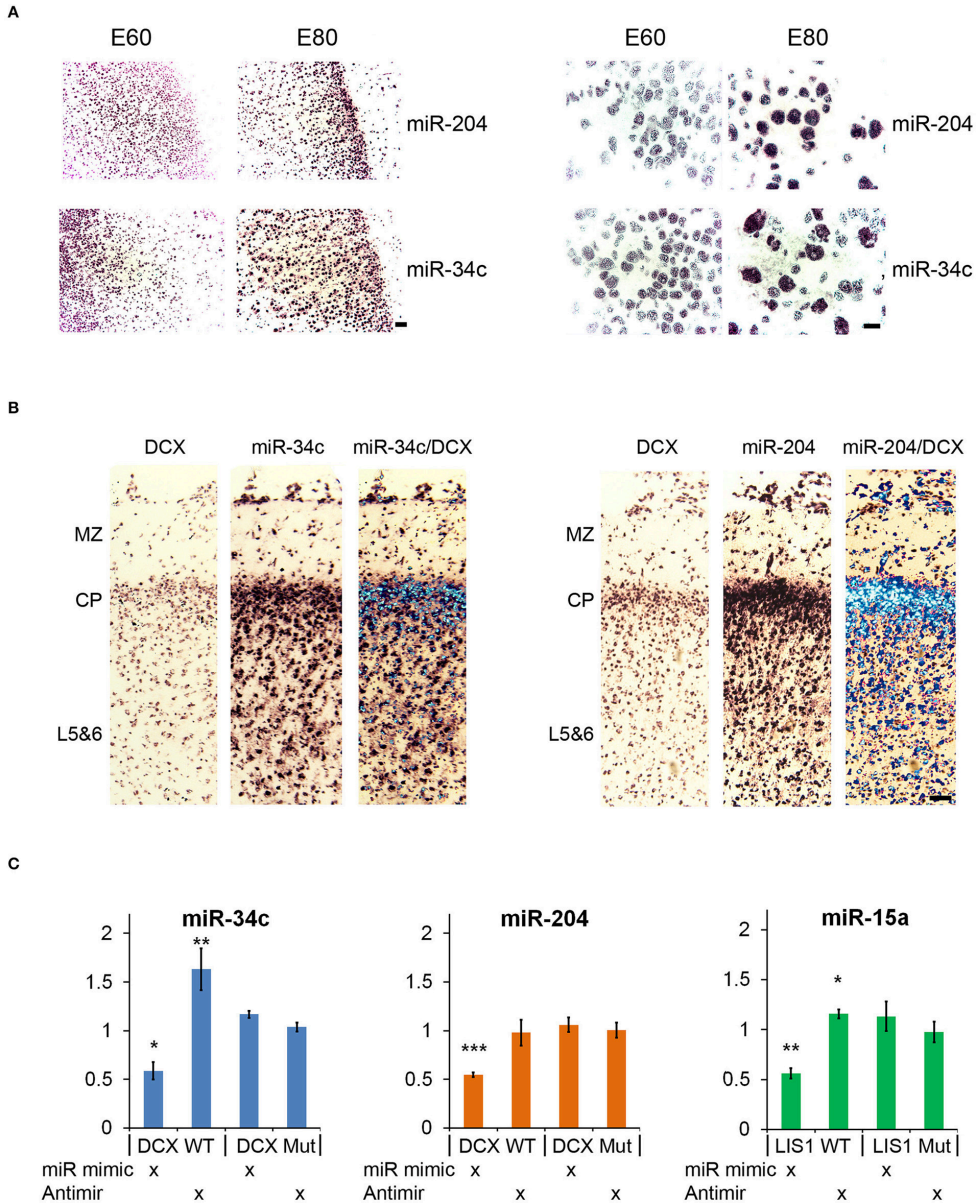
**In situ hybridization and luciferase reporter assay. (A)** *In situ* hybridization (ISH) images of miR-204 and miR-34c in E60 and E80 cortical porcine tissue. Upregulation from E60 to E80 is evident for both miRNAs. Scale bars: 50 mm (left panel), 10 mm (right panel). **(B)** Co-localization of DCX with miR-34c and miR-204 is shown by merged ISH signals as indicated. MZ, marginal zone; CP, cortical plate; L5&6, layer 5-6. Scale bar: 50 mm. **(C)** Luciferase 3′UTR-reporter assays. miR-34c and miR-204 mimics are shown to reduce DCX 3′UTR luciferase reporter expression, and miR-34c inhibitor increases DCX 3′UTR luciferase reporter expression. MiR-15 mimic reduces LIS1 3′UTR luciferase reporter expression, while miR-15 inhibitor slightly increases its expression. Luciferase data is normalized to relevant control (control mimic or control inhibitor), and experiments were done in biological triplicate. Data are presented as means ± standard deviation. ^*^P < 0.05, ^**^P < 0.01, ^***^P < 0.001. Independent Sample *T*-test.

Small RNA sequencing of embryonic porcine cortex across fetal development revealed a remarkable shift in expression levels of a distinct set of miRNAs from embryonic day 60–80 (E60–E80), which is the period of cortical folding in the pig brain (manuscript in preparation). In total 49 miRNAs, expressed at least 100 reads per million (RPM), exhibited an at least two-fold change in expression in cortex from E60 to E80 (45 up- and 4 down-regulated). Restricting them to conserved miRNA families reduced the number of regulated miRNAs to 32 up- and 3 downregulated (Table 1). Conserved 3′UTR targets of this set of miRNAs were detected using TargetScan version 7 (Agarwal et al., 2015). Global miRNA targeting analysis (see Section Experimental Procedures) of the differentially expressed miRNAs revealed a list of genes predicted to experience altered miRNA-mediated control from E60 to E80 (Supplementary Table 1). Among the top 5 most significantly affected genes, DCX is of particular interest since it has been implicated in migration of cortical neurons, and hence is a prime target for miRNA-mediated control in respect to cortical morphogenesis. TargetScan predicts that 14 out of 32 (40%) upregulated miRNAs target DCX. Furthermore, none of the three downregulated miRNAs target DCX, whereas all three target LIS1 (Figures 1A,C; Table 1). In agreement with the predicted targeting of the differentially expressed miRNAs, DCX, and LIS1 are down and upregulated, respectively, upon gyration (Nielsen et al., 2010). MiR-204 and miR-34c are particularly notable upregulated miRNAs, due their almost exclusive expression in E80 cortex tissue (Figure 1B). This encouraged us to examine these miRNAs further. Detection of miR-204 and miR-34c by *in situ* hybridization (ISH) at E60 and E80 in the porcine cortex showed clearly increased expression at E80 for both miRNAs (Figure 2A). No expression difference was observed between sulci and gyri (Supplementary Figure 1).

**Table 1:** **Differentially expressed conserved miRNAs**.

**miRNA**	**60Cx - RPM**	**80Cx - RPM**	**Fold change**	**TargetScan7 family**	**No. dcx targets**	**No. lis1 targets**
ssc-miR-10a-5p	35.3	165.9	4.7	miR-10-5p	0	0
ssc-miR-10b	230.1	957	4.2	miR-10-5p	0	0
ssc-miR-126-3p	434.6	1650.5	3.8	miR-126-3p.1	0	0
ssc-miR-129a-5p	30.7	144.2	4.7	miR-129-5p	1	0
ssc-miR-132	109	292.6	2.7	miR-132-3p/212-3p	0	0
ssc-miR-142-5p	47.2	130.4	2.8	miR-142-5p/5590-3p	0	0
ssc-miR-143-3p	8455.7	30879.9	3.7	miR-143-3p/4770/6088	1	0
ssc-miR-148a-3p	4189.3	12959.3	3.1	miR-148-3p/152-3p	0	0
ssc-miR-152	60.8	229.4	3.8	miR-148-3p/152-3p	0	0
ssc-miR-424-5p	20.3	106.8	5.2	miR-15-5p/16-5p/195-5p/424-5p/497-5p/6838-5p	0	3
ssc-miR-199a-3p	392.9	1486	3.8	miR-199-3p/3129-5p	0	0
ssc-miR-199a-5p	49.6	265.8	5.3	miR-199-5p	0	0
ssc-miR-204	361.8	41785.8	115.5	miR-204-5p/211-5p	2	0
ssc-miR-208b	11.3	150.7	13.3	miR-208-3p	0	0
ssc-miR-210	46.6	107.6	2.3	miR-210-3p	0	0
ssc-miR-218-5p	47.2	127.3	2.7	miR-218-5p	1	0
ssc-miR-23a	87.9	290.4	3.3	miR-23-3p/130a-5p	0	0
ssc-miR-24-3p	309.8	891.8	2.9	miR-24-3p	0	0
ssc-miR-363	874.3	1927.9	2.2	miR-25-3p/32-5p/92-3p/363-3p/367-3p	1	1
ssc-miR-92b-3p	5112.3	16944.1	3.3	miR-25-3p/32-5p/92-3p/363-3p/367-3p	1	1
ssc-miR-27a	168.7	545.1	3.2	miR-27-3p	2	0
ssc-miR-30a-5p	3517.8	8437.5	2.4	miR-30-5p	1	0
ssc-miR-30b-5p	179.3	771.6	4.3	miR-30-5p	1	0
ssc-miR-30c-5p	534.7	1612.1	3	miR-30-5p	1	0
ssc-miR-31	35.8	130.4	3.6	miR-31-5p	0	0
ssc-miR-328	30.7	104.5	3.4	miR-328-3p	0	0
ssc-miR-34a	2.3	122.5	50.2	miR-34-5p/449-5p	1	0
ssc-miR-34c	75.4	13591.4	180.1	miR-34-5p/449-5p	1	0
ssc-miR-378	320.8	989.3	3.1	miR-378-3p/422	1	0
ssc-miR-451	94.6	536.5	5.7	miR-451a	0	0
ssc-miR-486	1458.6	3540.6	2.4	miR-486-5p	0	0
ssc-miR-542-3p	15	183	12.1	miR-542-3p	1	0
ssc-miR-15a	746.7	344.9	−2.2	miR-15-5p/16-5p/195-5p/424-5p/497-5p/6838-5p	0	3
ssc-miR-16	15528.7	6901.3	−2.3	miR-15-5p/16-5p/195-5p/424-5p/497-5p/6838-5p	0	3
ssc-miR-17-5p	378.9	171.2	−2.2	miR-17-5p/20-5p/93-5p/106-5p/519-3p/526-3p	0	1

*All differentially expressed conserved miRNAs in porcine cortex at E60-E80*.

### Validation of miRNA targeting

Both miR-34c and miR-204 are predicted by TargetScan to target DCX mRNA through 1 and 2 target sites, respectively (Table 1). This functional connection was further substantiated by co-localization of both miRNAs and DCX mRNA at most cells in the cortical plate using ISH (Figure 2B). To confirm that the predicted target sites are indeed functional targets of miR-34c and miR-204, the DCX 3′UTR was cloned downstream of a luciferase reporter and analyzed in HEK293-H cells. In accordance with being cognate targets, mimics of both miR-204 and mir-34c were able to significantly reduce luciferase expression, whereas miR-34c inhibitor increased the production from the DCX 3′UTR reporter. However, we were unable to observe any effect from miR-204 anti-miR (Figure 2C). The most prominent LIS1 targeting miRNA family contained two downregulated miRNAs, miR-15a and miR-16, with three predicted targets in the LIS1 3′UTR (Table 1). Choosing miR-15a as a representative for this miRNA family, we were able to confirm the predicted targeting, since miR-15a mimic and inhibitor reduced and increased luciferase expression from LIS1 3′UTR reporter, respectively. Mutating the predicted miR-34c, miR-204 and miR-15a target sites individually in the luciferase reporters abolished the observed effects (Figure 2C).

### miR-34c and miR-204 affect neuronal migration

**Figure 3:**
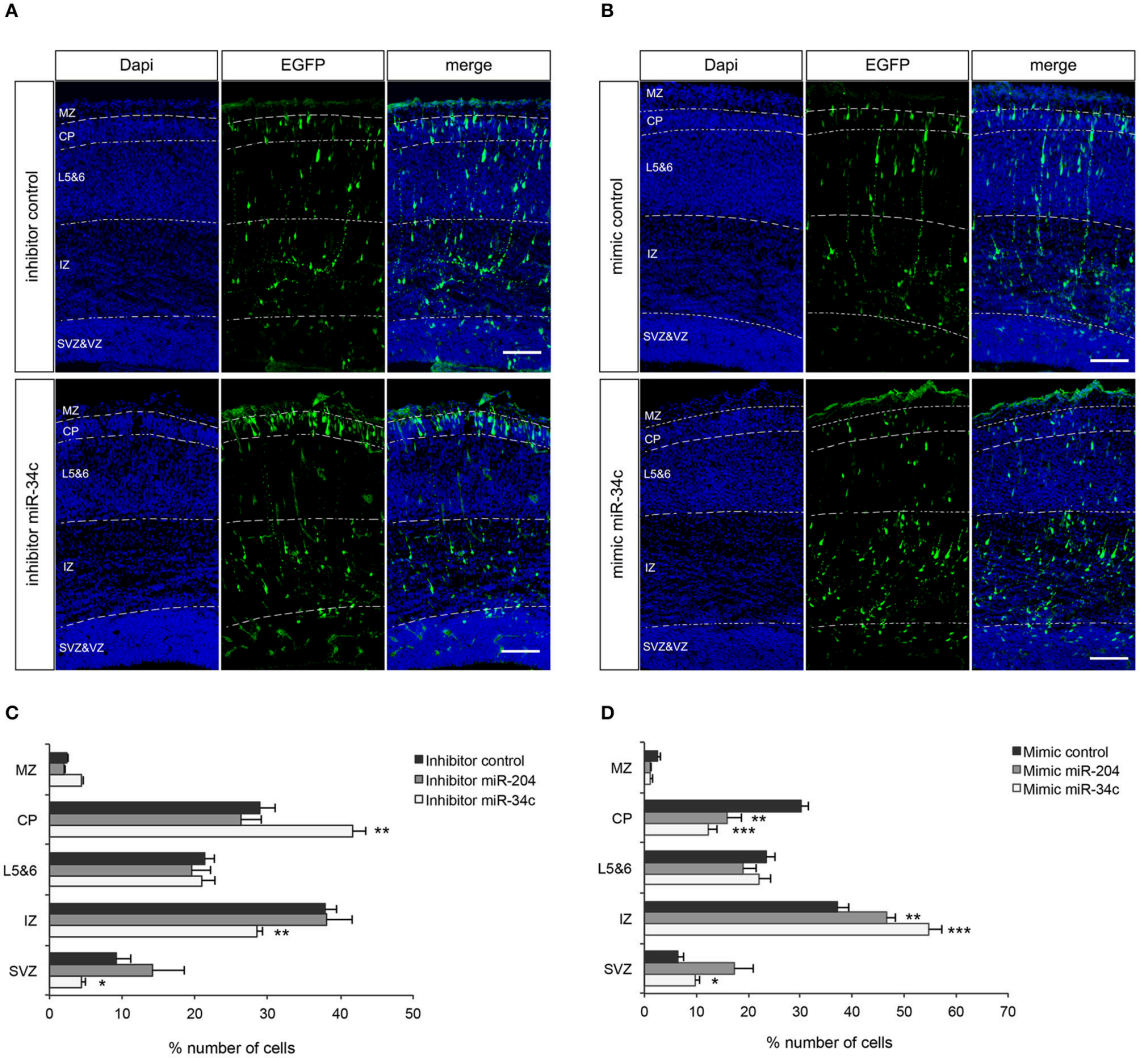
**miR-34c and miR-204 regulate cortical neuron migration. (A,B)** Coronal sections from E17.5 mouse embryos showing immunohistochemistry for GFP (green) and DAPI counterstaining (blue). Panels at the left show DAPI to indicate five different layers; MZ, marginal zone; CP, cortical plate; L5&6, layer 5-6; IZ, intermediate zone; and SVZ, subventricular zone. GFP signal indicates neurons transfected with miR-34c inhibitor **(A)** or mimic **(B)**. Representative miR-204 experiment is in Supplementary Figure 2. **(C,D)** Quantification of GFP-positive cells (% of total) in different layers. Data are presented as means ± SEM. ^*^P < 0.05, ^**^P < 0.01, ^***^P < 0.001. Independent Sample *T*-test. Number of embryos analyzed: miR-34c inhibitor: 5, miR-34c mimic: 5, miR-204 inhibitor: 6, miR-204 mimic: 7, control inhibitor: 9, control mimic: 8.

Both miR-34c and miR-204 are conserved between mouse and pig and despite the fact that mice have a non-gyrated brain, the underlying process of neuronal migration is conserved between lissencephalic and gyrencephalic species (Kerjan and Gleeson, 2007). Hence, to explore the function of these miRNAs *in vivo*, miRNA mimics or inhibitors together with a GFP expressing plasmid were introduced to neuronal progenitors in the ventricular zone of the cortex by *in utero* electroporation (IUE) in mouse fetus at E14.5 followed by immunohistochemical characterization of the neurons that derived from these progenitors at E17.5 (van Erp et al., 2015). Analysis of migration of GFP-positive neurons in the motor cortex revealed that inhibition of miR-34c induced a robust increase in the percentage of GFP-positive cells in the cortical plate (CP; *P* = 0.006) and a reduction in both the intermediate zone (IZ; *P* = 0.007) and the subventricular zone (SVZ; *P* = 0.042) (Figures 3A,C). Electroporation of miR-204 inhibitor did not significantly alter the radial migration of GFP-positive cells (Figure 3C). In contrast, electroporation of miR-34c or miR-204 mimics caused cells to accumulate in the IZ (miR-34c; *P* = 0.001, miR-204; *P* = 0.004) and resulted in reduced cell numbers in the cortical plate (miR-34c; *P* = 0.000, miR-204 *P* = 0.002) compared to control mimic (Figures 3B,D). Together, these data strongly suggest that miR-34c and miR-204 regulate neuronal migration in the embryonic cortex, in line with our expression and reporter assay experiments.

## Discussion

Cortical folding is a complex process orchestrated by spatially controlled gene expression in subsections of the brain, and miRNA is likely to play key roles in the process. MiR-204 and miR-34c sequences and their predicted targets in DCX 3′UTR are conserved, implying that the effects of miR-34c and miR-204 on the migration of neurons in the mouse is most likely conserved in higher mammals, such as pigs and humans. Further support for a conserved functional relationships between DCX and miR-204/34c as well as LIS1 and miR-15a comes from the observation that the respective miRNAs can regulate luciferase expression under control of human derived DCX and LIS1 3′UTRs.

The striking finding that miR-204 and miR-34c exhibit specific expression increases in excess of 100 fold at the time of porcine gyration implies that they have key roles in the timing of the process. Notably, these two miRNAs alone change from low expression to constituting a massive 5.5% of all cortical miRNAs during gyration. miR-204 shares an identical seed sequence with mR-211 in most animals, however, this miRNA is not annotated in pig, and thus is not found in the analysis of our sequencing experiment. A recent paper reported that inactivation of two miRNA clusters, miR-34b/c and miR-449 clusters, with identical seed sequences, affected brain development, and microtubule dynamics (Wu et al., 2014). These findings are well in line with our data. The authors also find DCX to be a direct target of the miR-34-5p/449-5p family. Likewise, miR-34a has a documented role in neural cell differentiation and migration of neuroblasts, suggested to be caused by targeting of DCX (Mollinari et al., 2015). Altogether, every member of the miR-34-5p/449-5p family of miRNAs, all sharing identical seed sequences, have been found to affect brain development. Of the 6 miRNAs in this miRNA seed family (miR-34a,b,c, and miR-449a,b,c) only miR-34a and miR-34c are annotated in pig, in agreement with the detected miRNAs in our profiling experiment. Table 1 supports the shared function of miR-34a and miR-34c, given that both miRNAs show large expression increases from E60 to E80 of 50 and 180-fold, respectively. Although miR-34a and miR-34c have identical seed sequences, they have a substantial 5 nt sequence dissimilarity outside the seed, ensuring that miR-34a and miR-34c can readily be distinguished in ISH experiments via specific LNA probes.

Our finding of tightly controlled expression of miR-34c and miR-204 in brain development seems to extrapolate into neuroprotective effects in the aging animal. In a study by Liu et al. (2012), Drosophila miR-34 was found to display increased brain expression, specifically in old animals. The authors found that miR-34 over-expression ameliorated age-related neurodegeneration and increased median lifespan. This suggests that controlled upregulation of miR-34 family miRNAs is important, both in embryonic and adult animals.

Both the miR-34 family and miR-204 miRNAs are well known tumor suppressors in several cancers (He et al., 2007; Li et al., 2016) and have been linked to suppression of epithelial to mesenchymal transition (EMT; Hahn et al., 2013; Morizane et al., 2014; Li et al., 2016; Liu et al., 2016). In several cancers, including glioblastoma, metastasis is linked to the EMT process, whereby cancer cells can adopt mesenchymal traits allowing invasion into adjacent tissue (Ortensi et al., 2013). Initiation of neuronal migration is an EMT-like phenomenon, where cells lose epithelial characteristics and attain mesenchymal characteristics such as high motility (Itoh et al., 2013). Our finding, that increased miR-34c and miR-204 expression reduces neuronal migration, implies that suppression of EMT related factors could be a contributing mechanism controlling neuronal migration.

Temporal miRNA control during brain development is clearly important for animals later in life. DCX and LIS1, which our data suggest is under timing-specific miRNA-mediated control, are crucial for proper lamination of the brain. The known link between improper lamination and epilepsy (Stouffer et al., 2016) is highly relevant to our findings, since incorrect levels of both miR-34a and miR-204 have been associated with epilepsy (Hu et al., 2012; Kaalund et al., 2014; Xiang et al., 2016). Erroneous lamination in mouse brain caused by DCX knockout has been shown to result in increased seizure susceptibility and spontaneous epileptic seizures (Nosten-Bertrand et al., 2008), which the authors concluded was caused by abnormal synaptic transmission as a result of lamination defects. In humans, lissencephaly is tightly linked to epileptic seizures (Dobyns, 2010; Herbst et al., 2016), and focal cortical dysplasia (FCD), which involves some degree of disorganization of cortical lamination, is found in approximately half of the patients with medically refractory epilepsy (Wang et al., 2006). Thus, embryonic functions of miRNAs, such as miR-34c and miR-204, which affect cortical morphogenesis, are also likely to affect epilepsy susceptibility later in life.

Although we have shown that DCX is a target for both miR-34c and miR-204, and that over-expression of these miRNAs in IUE experiments significantly inhibit neuron migration in the mouse embryo, we cannot conclude that this migration effect is directly caused by inhibition of DCX. Given that single miRNAs can target multiple mRNAs, regulation of DCX may only be part of the picture. However, the strong genetic link between DCX and lissencephaly suggests that DCX may play a major role.

In summary, we show miRNA mediated control of DCX and LIS1 by miRNAs that are differentially expressed at the time of cortical folding. The repertoire of differentially expressed miRNAs at this developmental stage is likely to be crucial for timing the migration of neuronal precursors, thus affecting cortical morphogenesis. The most strongly upregulated miRNAs, miR-34c, and miR-204, were shown to alter the extent of neuronal migration in embryonic mouse brain, and their large expression increase during cortical folding in pig, suggests a pivotal role in mediating correct cortical morphogenesis of gyrencephalic animals.

### Experimental procedures

#### Illumina sequencing of miRNAs

All procedures involving pigs described in present study were reviewed and approved by the Danish Experimental Animal Inspectorate (“Rådet for Dyreforsøg”), Danish Ministry of Justice. Embryonic pig brain samples were snap frozen after dissection and transferred to RNAlater-ICE (Ambion). Low molecular weight RNA (<200 nt.) was purified using the MirVana kit (Ambion). Samples were DNase treated using the TURBO DNA-free Kit (Ambion). Library preparation was performed using the TruSeq Small RNA preparation kit (Illumina) using 100 ng low molecular weight RNA. Fifty bp single end sequencing was performed at Research Centre Foulum. One replicate was sequenced per timepoint. Raw sequencing data was quality filtered and trimmed using fastX toolkit and adapter trimmed using cutadapt. Filtered sequencing reads were mapped to the mature porcine miRNAs from miRBase version 21 using Bowtie. The resultant porcine miRNA expression profile was normalized to reads per million mapping reads (RPM).

#### Global miRNA targeting analysis

Conserved predicted targets of conserved miRNAs were downloaded from TargetScan version 7 (Agarwal et al., 2015). Unique miRNA family target sites were noted for all genes. The change in targeting was defined as number of matching upregulated miRNA families subtracting the number of downregulated miRNA families from E60 to E80. We observe 26 up- and 2 downregulated unique miRNA families in cortex from E60 to E80. To test significance, we randomly picked the same number of conserved miRNA families (100,000 times), revealing whether random picking generated equal or greater changes in targeting of matching miRNA families. *P*-values were generated for all expressed TargetScan indexed genes by dividing the number random occurrences with equal or greater targeting than observed by the number of iterations (100,000). Only expressed genes were assayed. Expression is here defined as minimum 1 FPKM in embryonic cortex from RNA-seq data presented in (Venø et al., 2015), GEO accession: GSE71832.

#### *In situ* hybridization

Brain slices (10 or 5 μm) from embryonic pigs (E60, E80; Venø et al., 2015) were *in situ* hybridized as previously described (Clausen et al., 2013). See Supplementary Table 2 for list of probes. The alkaline phosphatase-labeled probes used for miRNAs were 22 nts and 23 nts in length for miR-204 and miR-34c, respectively. The mRNA targeting alkaline phosphatase-labeled probes were 26 nts and 28 nts in length for GAPDH mRNA and DCX mRNA, respectively. Signal specificity was tested by hybridizing parallel sections with (1) 100-fold excess of unlabeled probe, (2) pre-treatment with RNase A (Pharmacia Biotech), or (3) buffer alone. All controls were devoid of signal.

The co-localization of miRNA with DCX mRNA was performed by hybridizing adjacent 5 μm thick sections with each probe, before overlaying the signals. The DCX mRNA and miRNA ISH probes were hybridized and developed for the same amount of time when performing co-localization experiments.

#### Toluidine blue staining

One series of brain sections from embryonic pigs (E60, E80) were stained with Toluidine Blue (Merck, Germany), dehydrated in graded series of alcohol (96–99% ethanol), cleared in xylene and cover-slipped in Depex (BDH Gurr, UK) as described (Clausen et al., 2006).

#### Luciferase reporter assay

Fragments of DCX or LIS1 3'UTRs were amplified by PCR using cDNA from HEK293-H. See Supplementary Table 2 for list of primers. The fragments were cloned into the psicheck2 vector and used for luciferase assay. Luciferase assay was performed by co-transfection of HEK293-H cells with miRNA mimic or inhibitor and psicheck2 reporter by standard calcium phosphate transfection. Three psicheck2 vectors with inserted fragment were made from WT sequences originating from:

DcxUp-3′UTR_WT: chrX:110543495-110543999       505 bp (The miR-34c target site).
DcxDown-3′UTR_WT: chrX:110537676-110538698   1023 bp (Both miR-204 target sites).
Lis1-3′UTR_WT: chr17:2586883-2588130                  1248 bp (All three miR-15a target sites).

Figure 1C shows the location from which the inserted fragments originate. Mutated versions of the three WT psicheck2 vectors were generated, carrying specific mutations in the miRNA target sites under investigation by use of Site-directed, Ligase-Independent Mutagenesis.

Cell lysates were harvested after 24 h and used for luciferase assay using the Dual-Luciferase® Reporter Assay System (promega) according to manufacture's recommendations.

#### *In utero* electroporation

All mouse use and care was carried out in accordance with institutional guidelines, Dutch law (Wet op de Dierproeven, 1996) and European regulation (Guideline 86/609/EEC), and with approval of the ethical animal experimentation committee (DEC). IUE was used to introduce miRNA mimics or inhibitors into the embryonic mouse cortex as described previously (van Erp et al., 2015). To visualize electroporated neurons, an EGFP expression vector was co-transfected with the mimics or inhibitors (pC1-EGFP and pCAG-EGFP both from Addgene). Electroporation was carried out at E14.5 according to an adapted protocol of (dal Maschio et al., 2012). Briefly, pregnant female mice were anesthetized with isofluorane (induction 5%; surgery 2,5%), their uterine horns were exposed, and 1.7 μl saline/Fast Green (0.3 mg/ml) mix solution containing 30 pmol RNA construct and 1.5 μg EGFP was microinjected into one of the lateral ventricles of each embryo. Embryos were electroporated with 5 impulses (30 V, 50 ms length and interval of 950 ms) using a tripolar electrode. At E17.5, embryonic brains were fixed overnight in 4% paraformaldehyde/PBS and subsequently cryoprotected in a 30% sucrose/PBS solution. Brains were sectioned at 20 μm (coronal) using a cryostat. Sections were immunostained using a rabbit anti-GFP antibody (1:1000, Thermo Fisher Scientific, Rockford, USA) overnight at 4°C (Schmidt et al., 2014). Sections were counterstained with DAPI and fluorescent staining was visualized on a Zeiss Axioskop A1 epifluorescent microscope. Neuronal migration was quantified as described previously (van Erp et al., 2015). In brief, the automated cell counting tool of ImageJ was used to determine the number of GFP-positive neurons in five different regions (marginal zone, cortical plate, layer 5–6, intermediate zone and SVZ). An independent sample *T*-test (SPSS software) was used to determine statistical significance.

## Author contributions

MTV conceived of the project. MTV and STV drafted the manuscript. IEH supplied embryonic porcine samples. MTV analyzed sequencing data and performed miRNA target analysis. STV performed luciferase assays. BHC and BF performed *in situ* hybridization. KR, JVA, and RJP performed and analyzed IUE. JK supervised the project.

### Conflict of interest statement

The authors declare that the research was conducted in the absence of any commercial or financial relationships that could be construed as a potential conflict of interest.
