# Supplementary figures and images for: Cortical Morphogenesis during Embryonic Development Is Regulated by miR-34c and miR-204
Source: Front Mol Neurosci. 2017 Feb 9;10:31. doi: 10.3389/fnmol.2017.00031 (PMC5299138; doi:10.3389/fnmol.2017.00031)

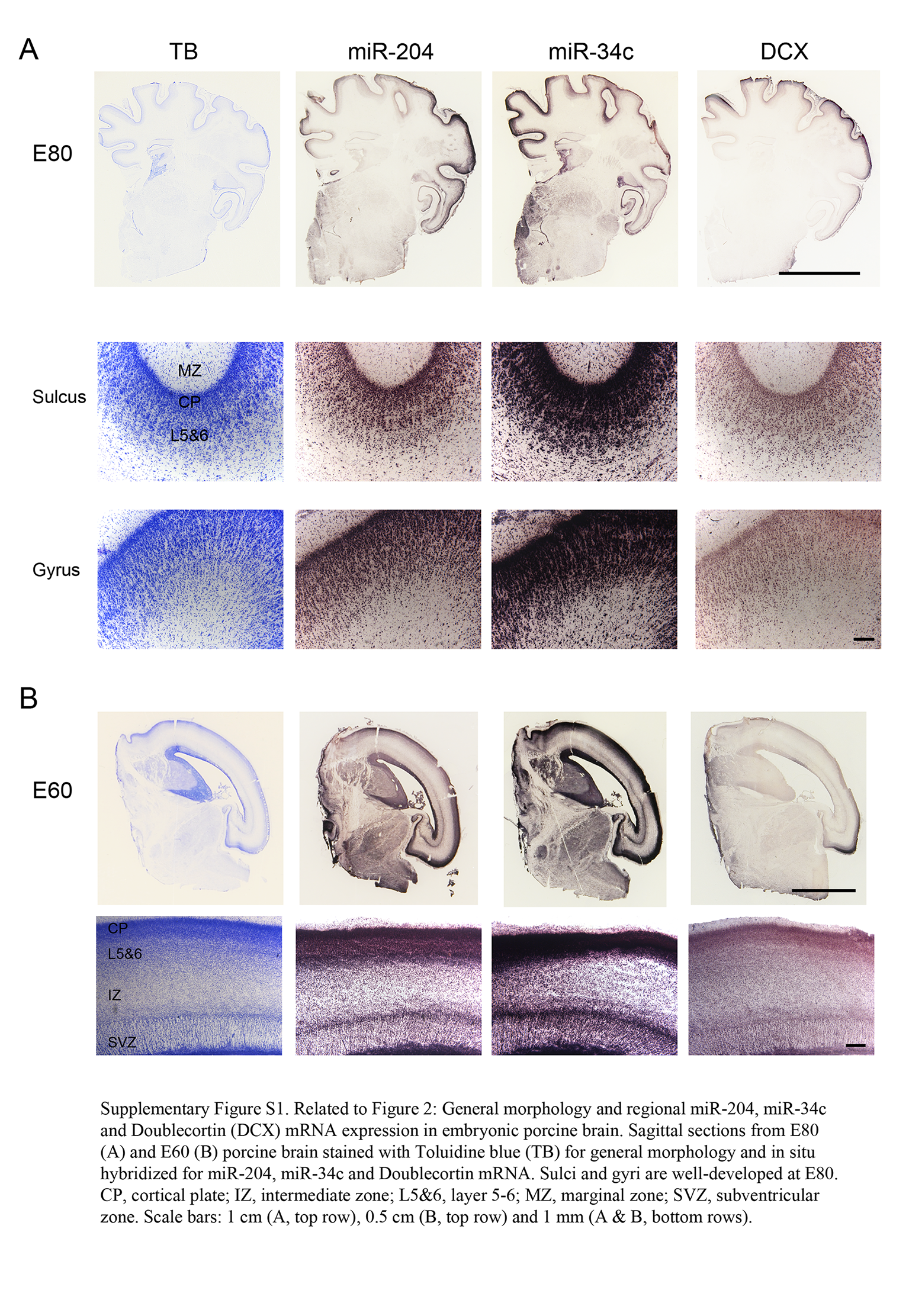

Supplement: Supplementary file 3 [file Image1.TIF]

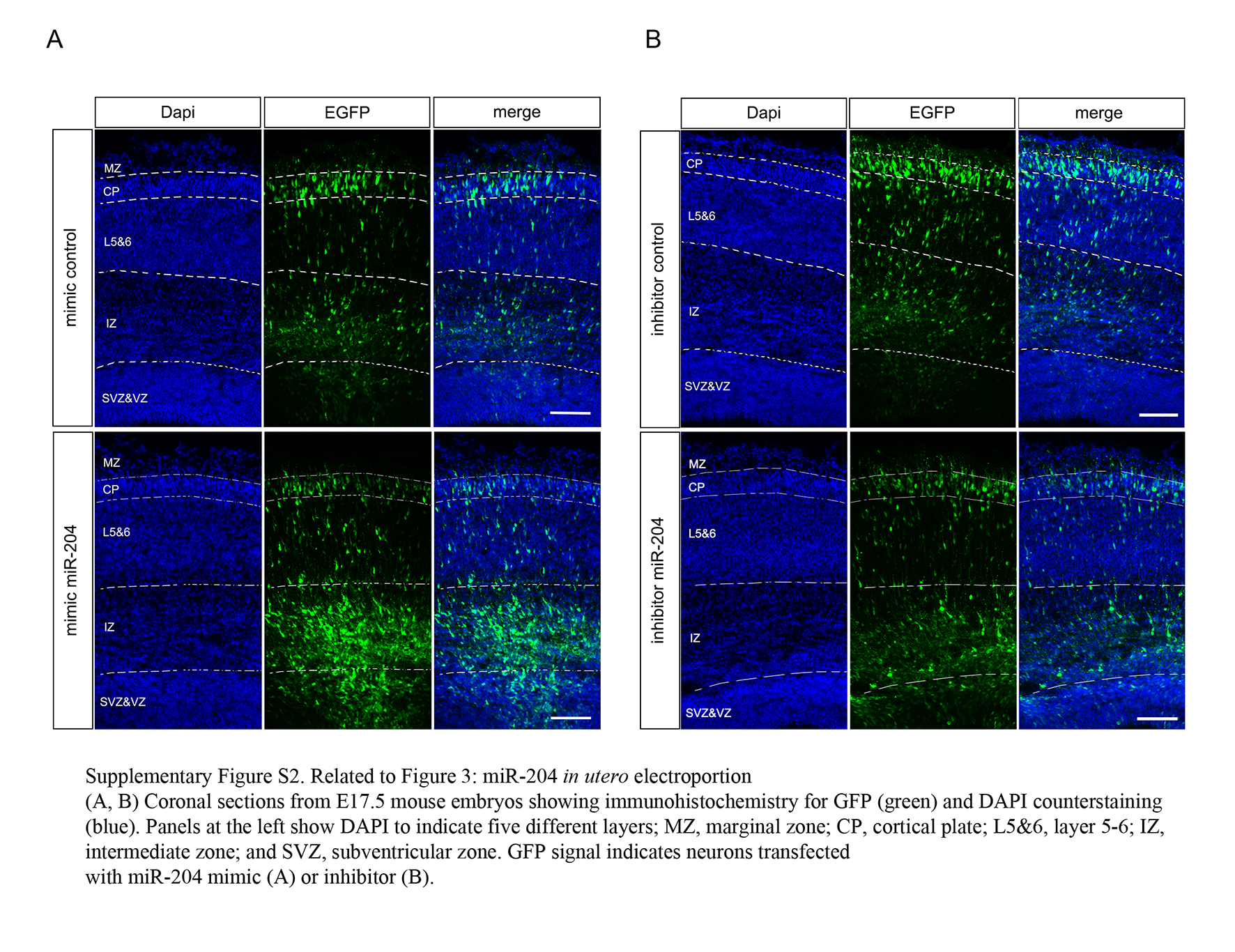

Supplement: Supplementary file 4 [file Image2.TIF]
